# Evaluating the Effects of the COVID-19 Pandemic on HIV Testing, Enrollment, ART Use and Mortality in Suriname Using Interrupted Time Series Analysis

**DOI:** 10.1007/s10461-025-04683-1

**Published:** 2025-04-10

**Authors:** Deborah Stijnberg, M. McKee, E. Commiesie, M. Adhin, W. Schrooten

**Affiliations:** 1 Faculty of Medicine and Life Sciences, Hasselt University, Hasselt, Belgium; 2 Faculty of Medical Sciences, Anton de Kom Universiteit van Suriname, Paramaribo, Suriname; 3 National AIDS Program, Ministry of Health, Paramaribo, Suriname; 4 National Tuberculosis Program, Ministry of Health, Paramaribo, Suriname

**Keywords:** COVID-19, HIV, Suriname

## Abstract

Our study evaluates the changes in HIV testing, new enrollments in the HIV surveillance system, treatment, and mortality of people with HIV during and after the SARS-CoV-2 (COVID-19) pandemic in Suriname. A retrospective population-based study was conducted, using interrupted time series analyses with data from the HIV surveillance system from January 2013 until December 2023. The commencement of the COVID-19 pandemic lead to a decline in HIV testing, enrollment, treatment initiation, and the annual number of individuals receiving treatment, respectively, by 16%, 32%, 40%, and 2% in 2020 compared to 2019. The mortality rate among people with HIV went from 7.8 in 2019 to 26 per 100,000 in 2022. The regression model showed an immediate significant effect at the start of the COVID pandemic for the HIV enrollments and the yearly number of people on treatment. For HIV mortality there is significant sustained effect. An overall decline in HIV services resulted in an increased mortality in 2021 and 2022. Innovative strategies and additional human and financial investments are needed to regain and improve access to health services and reverse the current epidemical trend.

## Introduction

On January 9th, 2020, the World Health Organization (WHO) reported that a novel coronavirus was the cause of the cases of pneumonia observed in Wuhan, China. By the end of January 2020, the WHO declared the outbreak a ‘public health emergency of international concern’ [1].

In Suriname, the first case of COVID-19 was diagnosed on March 13th, 2020. The government swiftly introduced different stringent public health interventions, such as the closing of borders and schools. Public transportation was also suspended indefinitely starting March 21st. By the end of March 2020, a partial lockdown was announced, and people were instructed to stay home from 8 at night until 6 in the morning. At that time, 10 imported cases were diagnosed in Suriname, and the first death was in April 2020. With the government elections on May 25th began the first wave of COVID-19. With the increase in cases, the number of people allowed in gatherings changed, local flights were suspended, areas were closed, casinos were closed, and schools remained closed. In June 2020, the country even faced a total lockdown for a month, allowing only use of essential services between 8 a.m. and 5 p.m. This was followed by periods of partial lockdowns [2], but public transport remained prohibited; this was only officially opened by the end of September 2020, when the first COVID-19 wave ended in Suriname [3]. In between, people depended on small illegal expensive buses or taxi.

With the introduction of COVID-19 in Suriname, medical services were also adjusted. The public was instructed to contact their family doctor, preferably by phone, especially if they had flu-like symptoms. After evaluation, the doctor could decide if the person should visit the clinic [4]. Different clinics established measures where patients would first be triaged at the door or window before entering for further examination. Additionally, in different hospitals, measures were in effect. COVID-19 care and treatment were upscaled, resulting in major downscaling of other essential health services. The polyclinic services of specialists were often reduced to (semi)urgent cases only, and staff (medical and non-medical) were redirected to other tasks and departments related to COVID-19 care [5]. In mid-December 2020, the second wave started, ending only in the first quarter of 2021.

During this pandemic, the WHO reiterated the importance of attaining universal health coverage, and while responding to the COVID-19 pandemic, other essential health services must be maintained. Initial assessments, which were based on reports from countries, revealed that, globally, 48% of countries had disruptions in essential primary care services, and 41% had disruptions in rehabilitative, palliative, and long-term care, which could affect the most vulnerable individuals, such as older persons and those living with chronic conditions [6]. Among the 128 countries reporting on communicable diseases, 36% mentioned disruptions in services related to infectious diseases such as HIV [6]. Although, initially, there was no data supporting more severe progression of COVID-19 in people with HIV (PWHIV) [7], more recent reports [8] from the WHO mention a 30% greater risk of severe or fatal COVID-19 for PWHIV. In any case, it was essential for antiretroviral treatment (ART) services to continue and be guaranteed [7, 8]. A modeling exercise from the WHO revealed that a 6-month interruption of ART could cause 500 000 extra deaths in sub-Saharan Africa alone [9]. In terms of HIV services worldwide, UNAIDS reported a decrease in HIV testing services in October 2020, although gaps in HIV treatment were found to be surprisingly less severe than initially feared [10]. Since then, different countries have reported some effects of COVID-19 on HIV services. Malawi, South Africa, and Guatemala reported decreases in HIV testing of 39%, 47.6% and 54.7%, respectively. In South Africa, ART initiation decreased 46.2%, whereas ART pick-ups remained stable [11]. In Malawi, referrals for HIV treatment ranged from 100 to 98.6% [12]. Guatemala caused an increase of 10.7% (*p* = 0.05) in deaths due to opportunistic infections (OIs), with a decrease in OI diagnoses of 43.7% (95% CI 41.0–46.2%) [13]. Little data regarding the effects of the COVID-19 pandemic on HIV services in the Caribbean and Latin America can be found.

Suriname sought to attain the UNAIDS targets of 90%, 81% and 73% of PWHIV who are aware of their HIV status, receive ART, and achieve viral suppression, respectively, by 2020. However, even before the COVID-19 pandemic, the country encountered difficulties in meeting these targets, demonstrating only 60%, 52% and 45% attainment in these categories in 2019 [14]. It is possible that the COVID-19 pandemic has exacerbated these challenges.

This study aimed to investigate the impact of measures taken during the COVID-19 pandemic on HIV testing, HIV treatment initiation, annual HIV treatment coverage, and mortality among PWHIV.

## Methods

### Study Design and Setting

A retrospective population-based study was conducted using HIV surveillance data collected from January 2013 until December 2023.

The Republic of Suriname, a multiethnic country, is geographically situated in South America and is culturally also part of the Caribbean. Suriname is divided into ten administrative districts, comprising two urban districts, six rural districts, and two interior districts that are challenging to access. Although Suriname is classified as an upper-middle-income country, the country is battling a recession [15]. HIV testing is widely available through voluntary counseling and testing (VCT) sites and private and hospital laboratories [16]. If they test positive, people should consult their family physician for enrolled in treatment and care. The national protocol states that treatment should be initiated for all PWHIV. Treatment is free of charge, but for other medical services, such as doctor visits and laboratory investigations, insurance or out-of-pocket payments are needed [16].

### Data Sources

The three sources of information used were the *national HIV testing database*, the HIV *Patient Master Index (PMI)* [17] version March 2024, and the *national mortality registration*. The *national HIV test database* contains information about HIV tests from different testing sites in the country. The test sites either have electronic or paper-based reporting with paper forms entered at the central level. The *HIV PMI* contains the national set of deduplicated unique codes of PWHIV, established through probabilistic (fuzzy) matching of HIV test, treatment, CD4/VL and the Prevention of Mother-to-Child-Transmission (PMTCT) data. Thus, a case-based surveillance system is created. Finally, *HIV mortality* data are reported to the Bureau of Public Health (BOG). BOG is the public health institute under the Ministry of Health responsible for the collection and analysis of data on all causes of death.

### Statistical Analysis

The indicators evaluated include the number of HIV tests performed, the number of newly enrolled individuals, ART initiation, the number of people yearly on treatment, and the number of deaths among individuals with HIV. The newly enrolled are all individuals seen for the first time with an HIV diagnosis in the PMI system. The yearly number of people on treatment are those with at least one ART pick-up in the year under consideration. Using descriptive statistics, the demographic data and summaries of the crude outcomes are presented. Where appropriate, the mean or median were calculated. Interrupted time series analysis was performed in R-Studio version 2023.12.1 + 402 [18] to compare results before, during and after the start of the COVID-19 pandemic for each indicator. This includes segmented regression analysis using the following formula:

1 $$Y_{{et}} = \beta _{0} + \beta _{1} T + \beta _{2} {\text{X}}_{t} + \beta _{3} {\text{TX}}_{t},$$

which represents the following [19]: Y_et_: the outcome at time *t*. *β*_0_: baseline at point T = 0 (2013). *β*_1_*T*: change in outcome per year before COVID-19. *β*_*2*_X_*t*_: change in outcome immediately after the start of the COVID-19 pandemic. *β*_*3*_TX_*t*_: sustained changes in outcome following the start of COVID-19.

Changes with p-values less than 0.05 were considered statistically significant. The Durbin-Watson test was performed to look at lag 1 autocorrelation. If autocorrelation was found, the differencing method, which looks at the differences between the yearly results, was executed.

## Results

### HIV Testing

**Fig. 1:**
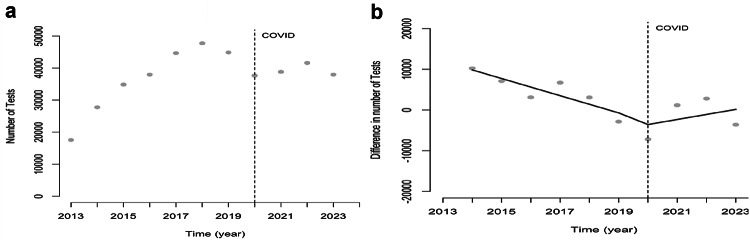
**a** Number of HIV tests conducted, 2013–2023. **b** Yearly difference in the Number of HIV tests performed with regression line pre, during and post start COVID epidemic in Suriname, 2013–2023. *Note* Vertical line indicates the start of the COVID 19 epidemic in Suriname in the first quarter of 2020

Prior to the COVID-19 pandemic, there was an annual increase in the number of HIV tests conducted, reaching a maximum of almost 48,000 tests in 2018. In 2019, there was a 6% decline, and in 2020, the year COVID-19 was introduced in Suriname, there was an additional drop of 16% (Fig. 1a). In the regression model, this shows as 12,529 less tests conducted (Table 1). Due to autocorrelation (DW test *p* = 0.0001), a regression model using differences in the yearly number of tests was performed (Fig. 1b). In this model, both the immediate and the sustained effects of the COVID-19 pandemic were not found to be significant (Table 2).

**Table 1:** Time series model for the yearly number of HIV tests performed pre, immediately at and post COVID in Suriname considering the period 2013–2023

	HIV testing	
	Estimate	95% CI
Pre-COVID	4704.4	(3078.1 to − 6330.7)*
COVID	− 12,528.7	(− 24,589.8 to − 467.5)*
Post COVID	− 4330.3	(− 8508.4 to − 152.2)*
Constant	22,365.8	(16,502.0 to 28,229.6)

*p < 0.05

**Table 2:** Time series model for the difference in the yearly number of HIV tests performed pre, immediately at and post COVID in Suriname considering the period 2013–2023

	HIV testing	
	Estimate of the difference	95% CI
Pre-COVID	− 2111.8	(− 4239.5 to 15,9444.9)
COVID	− 4099.2	(− 16,761.8 to 8563.4)
Post COVID	3351.4	(− 1162.2 to 7865.0)
Constant	11,945.5	(3659.1 to 20,231.8)

### Enrollment

**Fig. 2:**
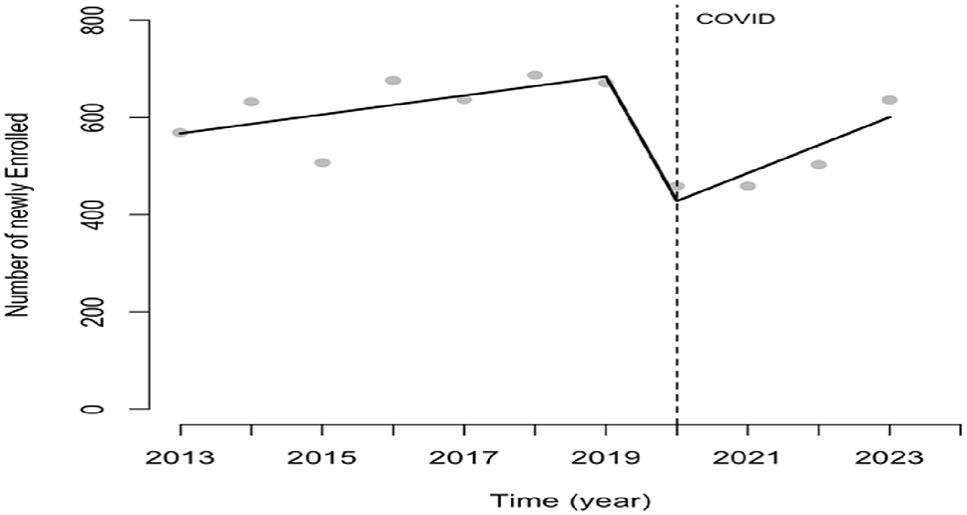
Yearly number of people newly enrolled with an HIV diagnosis with regression line pre, during and post start COVID epidemic in Suriname, 2013–2023. *Note* Vertical line indicates the start of the COVID 19 epidemic in Suriname in the first quarter of 2020

From 2013 to 2018, the number of newly enrolled patients fluctuated, with an average of 600 enrollments yearly, and a maximum of 687 new enrollments in 2018. The start of the COVID-19 pandemic decreased enrollments by 32% (Fig. 2). The regression model shows this as an immediate effect of 314 less enrollments (Table 3). This effect was not sustained in the post-COVID-19 introductory phase (Table 3).

**Table 3:** Time series model for the yearly number of people newly enrolled with an HIV diagnosis pre, immediately at and post COVID in Suriname considering the period 2013–2023

	HIV enrollment	
	Estimate	95% CI
Pre-COVID	19.5	(− 4.2 to 43.2)
COVID	− 313.6	(− 489.3 to − 137.8)*
Post COVID	38.0	(− 22.8 to 98.9)
Constant	567.1	(481.6 to 652.5)

*p < 0.05

### HIV Treatment Initiation

**Fig. 3:**
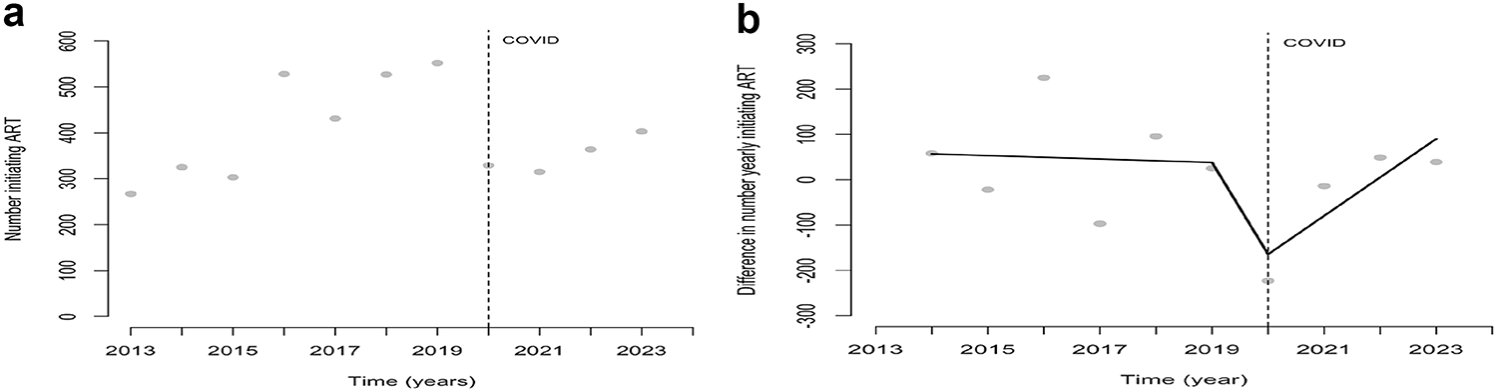
**a** The number of people initiating HIV treatment with regression line pre, during and post start COVID epidemic in Suriname, 2013–2023. **b** The yearly difference in the number of people initiating HIV treatment with regression line pre, during and post start COVID epidemic in Suriname, 2013–2023. *Note* Vertical line indicates the start of the COVID-19 epidemic in Suriname in the first quarter of 2020

During the study period, the percentage of people newly enrolled who were linked to care decreased from 84.7% in 2019 to an average of 77.8% in recent years. The number of PWHIV initiating treatment shows a 40% decrease, from 552 in 2019 to 329 in 2020. Time series analysis of the number of initiating treatments revealed autocorrelation (DW test *p* = 0.04), necessitating the completion of the regression model using the difference in enrollment between the years. In both models, no direct or sustained effects of COVID-19 were observed (Fig. 3; Tables 4 and 5).

**Table 4:** Time series model for the yearly number initiating HIV treatment pre, immediately at and post COVID introduction in Suriname considering the period 2013–2023

	Number initiating ART	
	Estimate of the number	95% CI
Pre-COVID	49.5	(26.2 to 72.8)*
COVID	− 282.6	(− 455.4 to − 109.8)*
Post COVID	− 22.4	(− 82.3 to 37.4)
Constant	270.4	(186.4 to 354.4)

*p < 0.05

**Table 5:** Time series model for the difference of yearly number initiating HIV treatment pre, immediately at and post COVID introduction in Suriname considering the period 2013–2023

	Number initiating ART	
	Estimate of the difference	95% CI
Pre-COVID	− 3.8	(− 67.9 to 60.4)
COVID	− 372.7	(− 669.3 to 94.3)
Post COVID	139.8	(− 47.4 to 224.8)
Constant	60.8	(− 189.1 to 310.7)

### Number of Persons on Treatment

**Fig. 4:**
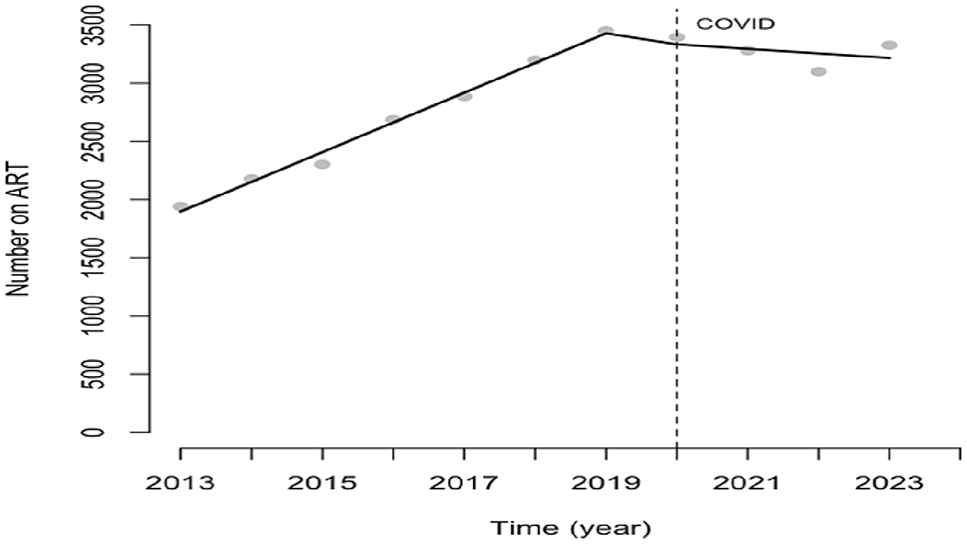
Yearly number of people on HIV treatment with regression line pre, during and post start COVID epidemic in Suriname, 2013–2023. *Note* Vertical line indicates the start of the COVID 19 epidemic in Suriname in the first quarter of 2020

From 2013 to 2019, the yearly number of persons receiving HIV treatment increased on average by 256 persons (Table 6). The year 2019 witnessed the highest number of people on ART, 3450. In 2020, a decline of 2% was noted in the number of people on treatment. In the post-COVID period, a sustained effect was observed, with on average 294 people less on treatment yearly (Fig. 4; Table 6).

**Table 6:** Time series model for the difference in the yearly number initiating HIV treatment pre, immediately at and post COVID introduction in Suriname considering the period 2013–2023

	Number on ART	
	Estimate of the number	95% CI
Pre-COVID	255.5	(215.2 to 295.8)*
COVID	− 56.5	(− 355.0 to 242.0)
Post COVID	− 294.3	(− 397.7 to − 190.9)*
Constant	1896.5	(1751.4 to 2041.5)

*p < 0.05

### Mortality in People with HIV

**Fig. 5:**
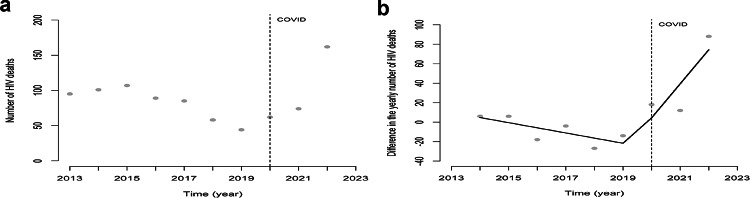
**a** The yearly number of people with HIV dying with regression line pre, during and post start COVID epidemic in Suriname, 2013–2022. **b** The difference in the yearly number of people with HIV dying with regression line pre, during and post start COVID epidemic in Suriname, 2013–2022. *Note* Vertical line indicates the start of the COVID 19 epidemic in Suriname in the first quarter of 2020

During this 10-year study period, 877 PWHIV died. In the pre-COVID-19 years, the HIV mortality rate declined, with a minimum of 7.8 per 100,000 in 2019 (Fig. 5a). The regression model for the number of people dying shows that this decline is significant (Table 7), but because of autocorrelation (*p* = 0.0086), the yearly difference model was used. In 2020, the number of people who died increased almost 1.5-fold, but this increase was not significant. Since 2021, the number of HIV-related deaths has significantly increased, with 40 more deaths every year (see Table 8; Fig. 5b). The mortality rate reached 26 per 100,000 in 2022.

**Table 7:** Time series model for the yearly number of HIV deaths pre, immediately at and post COVID introduction in Suriname considering the period 2013–2022

	Number of HIV deaths	
	Estimate of the difference	95% CI
Pre- COVID	− 5.3	(− 16.1 to 5.5)
COVID	− 8.9	(− 862.3 to 116.9)
Post COVID	40.3	(6.7 to 73.9)*
Constant	10.0	(− 31.9 to 51.9)

*p < 0.05

**Table 8:** Time series model for the difference in the yearly number of HIV deaths pre, immediately at and post COVID introduction in Suriname considering the period 2013–2022

	Number of HIV deaths	
	Estimate of the number	95% CI
Pre-COVID	− 9.3	(− 17.2 to − 1.4)*
COVID	− 55.4	(− 125.5 to 14.6)
Post COVID	59.3	(28.7 to 89.9)*
Constant	110.7	(82.1 to 139.2)

*p < 0.05

## Discussion

With the introduction of COVID-19 in Suriname in March 2020, yearly HIV testing, enrollment, treatment initiation and people on HIV treatment, decreased respectively with 16%, 32%, 40% and 2% in 2020 compared to 2019. While this difference was only significant for new enrollments, these declines represent a substantial setback in the already struggling HIV response in Suriname. In the post-COVID-19 period, a sustained decrease in the number of people on ART and an increase in the number of deaths were noted. The initial decrease in the number of tests seems less than globally reported. An overall decrease in testing of 48% is reported worldwide, whereas countries such as Malawi, South Africa and Guatemala report decreases of 39%, 48% and 55%, respectively [12, 13]. In the Latin America and Caribbean (LAC) countries, a decrease of 44.6% in tests and 56% in new care enrollments were reported [20]. The introduction of COVID-19 with accompanied regulatory interventions in March 2020 could explain the immediate decrease in the number of people testing, being diagnosed, and initiating treatment. Lockdowns, the cessation of public transport for several months and the limited availability of healthcare workers, many having to work in COVID-19 care, resulted in a decrease in the scale of non-COVID-19 health services. The disproportionate decrease in ART initiation compared with testing could be explained by the specialist services being even less accessible than primary care services. HIV testing is often initiated by primary care physicians, whereas ART services are more often provided by internal specialists, who, during the COVID-19 pandemic, were closely involved in COVID-19 care. A systematic review of ART disruptions mentioned fear of contracting COVID-19, decreased access to healthcare clinics, and decreased supply of ART in countries as some of the explanations [21]. These could all also be applicable in Suriname. Medication stockouts have persisted since 2020, which may account for the sustained effected on the annual number of individuals receiving treatment.

In different parts of the world, the role of nongovernmental organizations (NGOs) in maintaining services has been mentioned. In some European countries, where a decrease in testing has been observed, self-testing has increased with the availability of NGO 24-hour online counseling services. Even though clinic appointments were suspended because COVID-19 care took all resources, long-term medication was guaranteed [22]. This finding is in line with the WHO’s recommendation for the dispensing of multiple months of ART during the pandemic [23]. Self-testing is not yet fully implemented in Suriname, and acceleration of the implementation should be considered. This should be accompanied by a good system for linkage to care, as only 80% of people diagnosed in Suriname are linked to care [24]. While NGOs are already active in Suriname, their role in helping reduce the effects of the pandemic and increasing adherence needs to be scaled up and structured.

The increased HIV mortality is in line with different modeling studies mentioning an increase in mortality because of the COVID-19 pandemic. One study reported a 10% increase in mortality within 5 years in a high-prevalence country [25]. A study in Brazil reported 6.9% and 13.9% increases in mortality in 2020 and 2021, respectively, and some cities even reported more than 87% increase [26]. The increase in deaths is related to ART interruptions, but other explanations should also be considered.

This highlights the first limitation of this study. Other potential factors explaining these results, such as economic and HIV program recessions during this period, are not considered. The National AIDS Program dealt with stockouts in tests, medication, and commodities in the last 3 years, and there were limited interventions implemented to decrease the HIV burden [27]. This amplified the effect of the COVID-19 pandemic. Another limitation is the limited number of data points post-COVID-19, which restricts a robust regression model. This could explain why no significant differences were found even at the beginning of the COVID-19 pandemic when all the indicators evaluated worsened. The national character of the data and the fact that different measures are evaluated are some of the strengths of this study. The immediate changes noted in all these measures still point to the effect of COVID-19 on the HIV situation in Suriname.

This study revealed an immediate decrease in the number of HIV tests performed, new enrollments, treatment initiations and people on ART with the start of the COVID-19 pandemic in March 2020 in Suriname. It has not been proven that this can be attributed solely to the pandemic. Two years after COVID-19, with also a noticeable decline in the national HIV response, the number of people on ART is still decreased, and the number of deaths, among people with HIV, has increased. As ART is a cornerstone of the HIV response, it is important to introduce innovative approaches such as digital options to optimize ART services. In all situations, as recommended by the World Health Organization, securing ART for people with HIV, remains essential. The government of Suriname, together with different stakeholders, need to scale up innovative interventions to reverse the current trend of the HIV epidemic in Suriname.

## Data Availability

Because of confidentiality, the data is available on request and after approval by the Ministry of Health Suriname.

## Code Availability

Available on request.
